# Clinical implications of fracture-associated vascular damage in extremity and pelvic trauma

**DOI:** 10.1186/s12891-018-2333-y

**Published:** 2018-11-20

**Authors:** F. Gilbert, C. Schneemann, C. J. Scholz, R. Kickuth, R. H. Meffert, R. Wildenauer, U. Lorenz, R. Kellersmann, A. Busch

**Affiliations:** 1 Department of Orthopaedic Trauma, Hand, Plastic and Reconstructive Surgery, University Hospital Würzburg, Würzburg, Germany; 2 Department for General Visceral, Vascular & Paediatric Surgery, University Hospital Würzburg, Würzburg, Germany; 3 Core Unit Systems Medicine IZKF, University Hospital Würzburg, Würzburg, Germany; 4 Department of Diagnostic and Interventional Radiology, University Hospital Würzburg, Würzburg, Germany; 5 Department of Vascular Surgery, Klinikum Fulda, Fulda, Germany; 6 Department for Vascular and Endovascular Surgery Klinikum rechts der Isar, Technical University Munich, Munich, Germany; 7 Department of Trauma Hand Plastic and Reconstructive Surgery, University Munich Germany, Julius-Maximilians-University of Würzburg Oberdürrbacherstr, 6 D-, 97080 Würzburg, Germany

**Keywords:** Fracture-associated vascular damage, Surgical trauma room, Extremity trauma, Pelvic trauma, Endovascular repair, Level of evidence: IV

## Abstract

**Background:**

Vascular damage in polytrauma patients is associated with high mortality and morbidity. Therefore, specific clinical implications of vascular damage with fractures in major trauma patients are reassessed.

**Methods:**

This comprehensive nine-year retrospective single center cohort study analyzed demography, laboratory, treatment and outcome data from 3689 patients, 64 patients with fracture-associated vascular injuries were identified and were compared to a control group.

Results: Vascular damage occurred in 7% of patients with upper and lower limb and pelvic fractures admitted to the trauma room. Overall survival was 80% in pelvic fracture and 97% in extremity fracture patients and comparable to non-vascular trauma patients. Additional arterial damage required substantial fluid administration and was visible as significantly anemia and disturbed coagulation tests upon admission. Open procedures were done in over 80% of peripheral extremity vascular damage. Endovascular procedures were predominant (87%) in pelvic injury.

**Conclusion:**

Vascular damage is associated with high mortality rates especially in combination with pelvic fractures. Initial anemia, disturbed coagulation tests and the need for extensive pre-clinical fluid substitution were observed in the cohort with vascular damage. Therefore, fast diagnosis and early interventional and surgical procedures are necessary to optimize patient-specific outcome.

**Electronic supplementary material:**

The online version of this article (10.1186/s12891-018-2333-y) contains supplementary material, which is available to authorized users.

## Background

Trauma is one of the leading causes of death in western countries, and the most frequent one in people below forty [1]. Whenever the body’s integrity is compromised by contusion, concussion or fracture, surgical trauma care is necessary for rapid damage control and specific individualized treatment. This remains, a major challenge for clinicians due to varying patterns of injuries and occasional major traumas, requiring urgent, yet highly specialized therapy [2, 3]. Fast, conclusive and complete injury assessment by examination of head and neck, thorax, abdomen, pelvis and the extremities is further supported by computed tomography (CT) and CT-angiography (CTA) [4, 5].

Head and thoracic injuries are found in approximately 50% of all trauma patients and extremity or pelvic fractures in roughly 30% [6]. Additional vascular damage, especially of arteries or venous plexus is acutely life threatening and further reduces time for decision-making and treatment [7]. Vascular damage in combination with fractures is associated with higher mortality and inferior outcome, especially due to rapid and voluminous blood loss into pelvic or femoral soft tissue compartments. Correct diagnosis may be difficult due to low body temperature and masking effects of other injuries [8–10]. Doppler ultrasound and CTA increase the detection rate, yet incorrect diagnosis remains frequent thus requiring re-examination after initial stabilization [11, 12]. Depending on type and extent of trauma, presentations may be acute bleeding, hemodynamic instability, pulsating tumors, massive hematoma or ischemic and pulseless extremities [13]. Additionally, vasospasm or complete vascular disruption both may mimic ischemia [14].

Despite recognized difficulties in detecting vascular injury in extremity trauma as well as an increased mortality rate in combination with pelvic trauma, limited data about the immediate clinical implications of vascular damage associated with fractures is available. We therefore analyzed thoroughly demography, initial laboratory results, treatment and outcome of extremity and pelvic fracture-associated vascular injuries in a trauma cohort of 64 patients versus a comparable cohort of patients with isolated osseous damage in a nine-year retrospective single-center study was carried out.

Aim of the study:

The aim of this retrospective cohort study was to recognize patterns of injury where vascular damage and fracture occur and also identification of surrogate parameters which may suspect a vascular involvement in major trauma patients. In addition, the influences of concomitant vascular damage to patient’s management and outcome were analyzed.

## Methods

### Facility

A university hospital and level-one trauma center, embedded in the regional trauma network (Traumanetzwerk Nordbayern-Würzburg) was analyzed. A multidisciplinary team provides twenty-four-seven trauma care; following standard operating procedures according to guidelines of the German National Society for Trauma Surgery (DGU) are implemented. Ultrasound, X-ray and multi-slice computed tomography (CT) are available on site in the trauma room. A board-certified vascular surgeon and an interventional radiologist are part of the trauma team.

Patient identification.

A retrospective analysis of all admissions to the surgical trauma room from December 2005 until December 2013 identified 3689 cases, including primary trauma admissions and secondary transfers from other centers.

The initial analysis included trauma mechanism and pattern of injuries, specifically identifying all vascular damages. Additional examination included fractures, immediate vascular, visceral, urologic or pediatric surgery or invasive vascular diagnostics and interventions. Every case with vascular involvement and extremity or pelvic fracture was then analyzed for age, sex, blunt or penetrating trauma, hospital stay, intensive care unit (ICU) stay, number of operations, method of treatment, amputation, death, trauma scores (Glasgow coma scale, GCS; injury severity index, ISS; Revised Injury Severity Classification Score, RISC I), preclinical fluid management and initial laboratory results. Data were available for 100% of patients, except for preclinical fluid management (36.7 and 49.3% for vascular injury group and fracture only group, respectively).

This cohort (vascular injury group) was then subdivided in regard to localization of the fracture, upper/lower extremity or pelvis, and the corresponding vascular damage. Patients with vascular involvement of the head and neck (*N* = 36), i.e. carotid artery dissection, were excluded in this study.

### Control group

In order to compare outcome and laboratory results, a control group consisting of 60 major trauma patients with limb/pelvic fractures only and no additional vascular damage, based on CTA, clinical assessment and clinical course, was selected. Inclusion criteria were surgical trauma room admission and fractures without further vascular, visceral, thoracic, spinal or cranial damage.

### Statistics

Group comparisons of count data were performed with χ2 tests when the smallest value in the contingency table was 5 or more, otherwise Fisher’s exact test was performed. Normally distributed measurement values (Shapiro-Wilk *p* ≥ 0.05) were examined with the Welch two sample t-test, in cases where the normality assumption did not hold (Shapiro-Wilk *p* < 0.05) the Wilcoxon rank sum test was applied. Dependencies between measurement values were examined with linear regression. Endpoint (survival) analyses were performed with Cox regression. *P*-values < 0.05 were considered significant.

## Results

### Demographic data

Within the nine-year study period, 3689 patients were admitted to the trauma room. Fractures of the extremities, the pelvis or the shoulder girdle (i.e. clavicula, scapula) were found in 904 patients. A third presented with multiple (≥2) fractures (Additional file 1: Table S1).

**Fig. 1:**
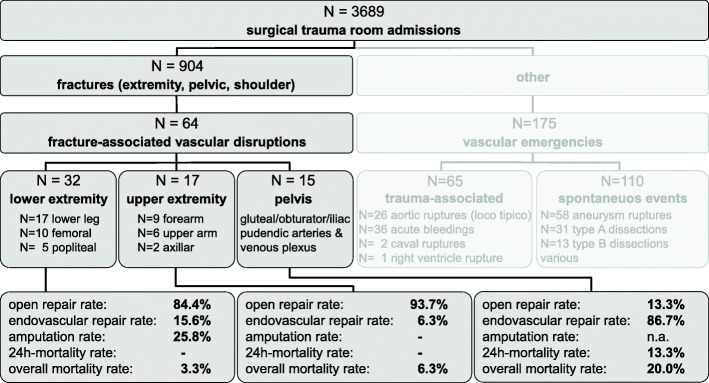
Demography, treatment and outcome of fracture-associated vascular injury. The chart shows the number (64 + 175 = 239) of vascular involvements in all 3689 surgical trauma room admissions in the study period (2005–2013). 64 cases of fracture-associated vascular disruption involving the upper/lower limb and the pelvis were treated by immediate open or endovascular repair. 175 cases required immediate vascular surgical exploration due to primary vascular emergencies like i.e. trauma-associated aortic rupture and acute bleeding or spontaneous aneurysm rupture or aortic dissection (blurred out)

**Fig. 2:**
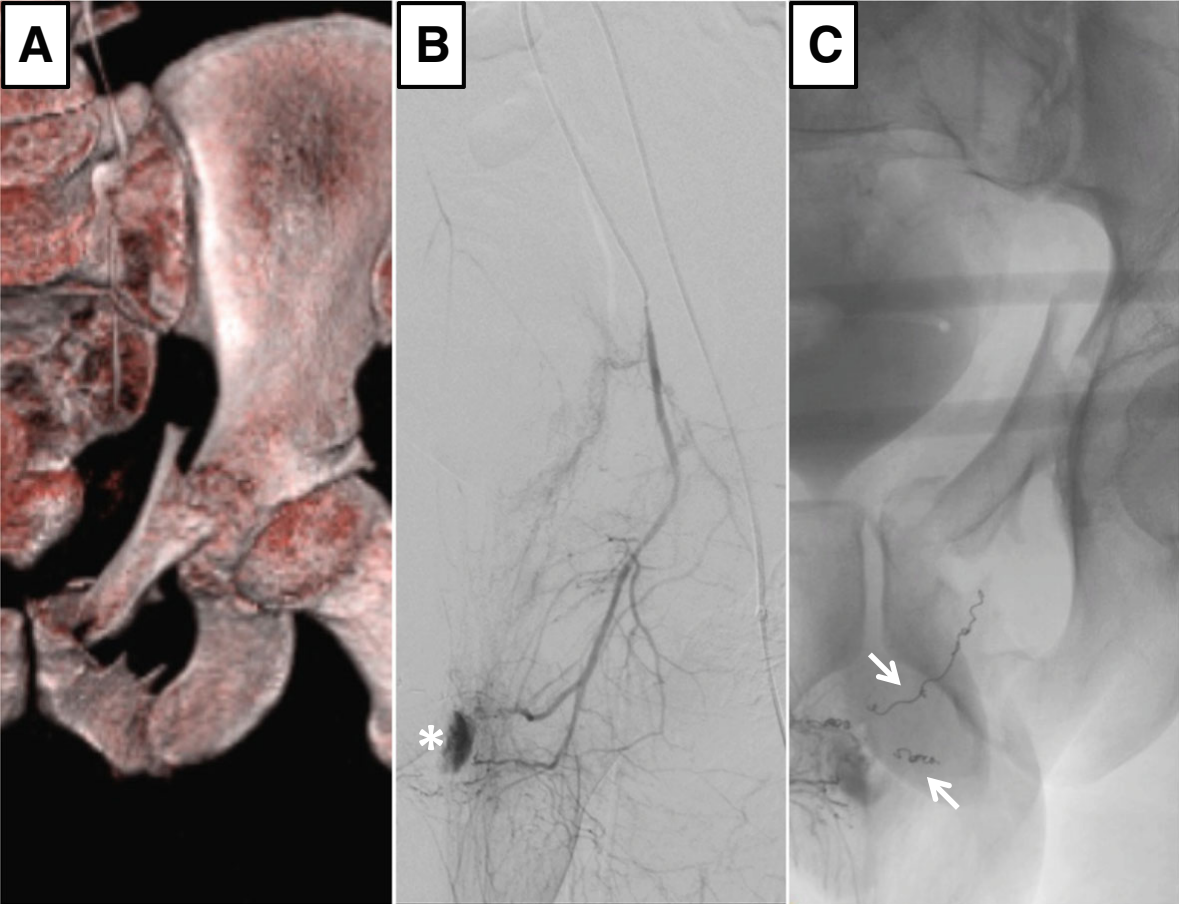
Pelvic fracture with associated arterial bleeding and consecutive embolization. The figure shows a 3D-reconstruction of a pelvic CT, with a type C pelvic fracture and destruction of the superior and inferior pubic rami at the time of trauma room admission in a hemodynamic unstable patient (**a**). CTA was suggestive of pelvic arterial bleeding and immediate angiography was indicated. Digital subtraction angiography (DSA) (**b**) revealed arterial bleeding from branches of the obturator artery with pooling of contrast agent around the symphysis (*). Super-selective microcoil embolization (arrows) of the feeding vessels helped to control the bleeding and stabilize the patient (**c**)

**Fig. 3:**
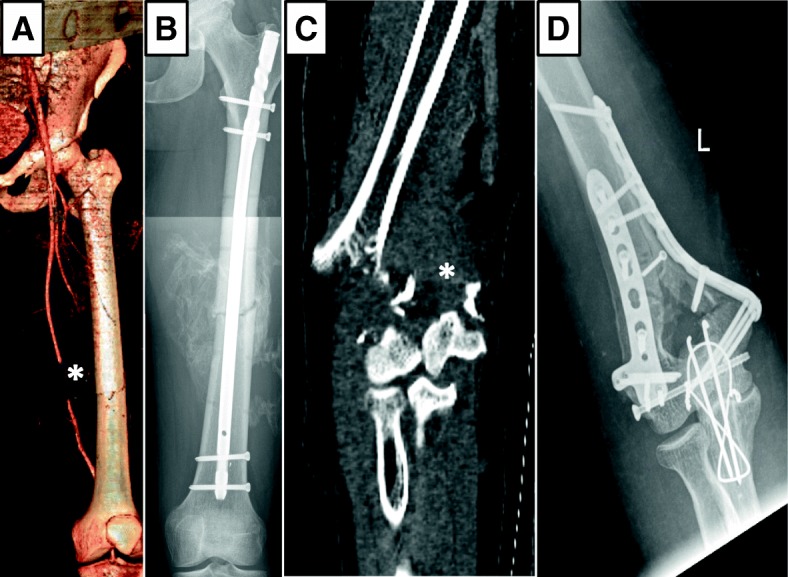
Extremity fractures with associated vascular damage. 3D-reconstruction (**a**) of the initial CT of a patient after motorcycle accident with closed femoral shaft fracture and disruption of the superficial femoral artery (★) with active bleeding palpable as pulsatile femoral mass (signal loss of soft tissue contrast agent pooling due to 3D-reconstruction sequence). Of note, fracture lines (**a**) indicate medial translocation of the osseous fragment towards the artery, probably causative of arterial rupture. Initial treatment included immediate open arterial reconstruction with vein graft interposition and external stabilization and after 12 days intramedullary nail repair (**b**). CTA with active pooling of contrast agent (*) from the brachial artery in a patient after fall with a grade IIIb open fracture of the humeral shaft (**c**). Initial treatment included patch plasty of the brachial artery, external fixation and fasciotomy of the forearm compartments. After 10 days the elbow joint was reconstructed with open reduction and plate fixation, an olecranon osteotomy was performed for better joint visualization

Vascular damage associated with a corresponding fracture of the extremities or the pelvis, requiring specific vascular repair, was identified in 64 cases (Fig. 1). Vessel injuries were confined to the arterial system in lower and upper limb, while arterial damage was the reason to treat in 66% in pelvic fractures and venous damage requiring treatment was seen in 34%. The mean age of this cohort was 49 ± 17 years, 78% were male and 94% sustained blunt trauma (79.4% traffic accident; 12.6% fall; 7.9% work-related accident) (Additional file 1: Table S1). The lower limb and its vessels were involved twice as often as upper limb and pelvis, however facture-associated vascular injury occurred at almost same rates in either localization (4.2, 6.2, 6.1% for upper limb, pelvis and lower limb, respectively) (Figs. 2 and 3). All patients had a CTA of the clinically suspected trauma area after exclusion of cerebral hemorrhage by native CT in cases of suspected head trauma. Depending on the treatment strategy, patients were then immediately transferred to the operating room or the angiography suite. A specific trauma mechanism could not be linked to vascular damage.

In total, 239 (6.5%) cases of all trauma room admissions had vascular implications that required immediate vascular repair (Fig. 1).

### Laboratory results

Comparing this group with a similar non-vascular trauma group, no differences in baseline characteristics were seen (Additional file 1: Table S1). However, fracture-associated arterial damage and consecutive blood loss into the surrounding soft tissues seriously aggravated the initial clinical presentation. Hemoglobin levels, a surrogate marker of blood loss, and prothrombin time (Quick) as well as activated partial thromboplastin time (PTT) were significantly lower in the vascular trauma group. Concordantly fibrinogen was at the lower limit (Table 1). These effects were pronounced in the pelvis and the lower extremity (Additional file 2: Table S2). Markers of cellular stress and disintegration, lactate, myoglobin and creatine kinase were elevated in both groups, yet showed no statistically significant difference (Table 1).

**Table 1:** Laboratory results and pre-clinical fluid administratio

	Hemoglobin (mg/dl)	Quick (%)	PTT (s)	Fibrinogen (g/l)	Lactate (mmol/l)	Myoglobin (μg/l)	Creatine kinase (U/l)	Crystallines (ml)	Colloids (ml)	Total volume (ml)
Vascular injury group	9.95 ± 2.6	59.2 ± 21.6	41.9 ± 25.2	1.7 ± 0.9	2.1 ± 1.2	1356 ± 1441	393 ± 316	1000 ± 741	500 ± 0	1500 ± 1112
Fracture only group	11.67 ± 2.6	78.0 ± 19.3	35.3 ± 20.7	2.2 ± 0.6	1.8 ± 0.9	855 ± 856	372 ± 337	1000 ± 0	500 ± 0	1000 ± 741
*P*	0.0004	0.0002	0.002	0.003	0.24	0.24	0.77	0.09	0.64	0.03

The table shows the mean ± standard deviation (SD) or median ± median absolute deviation (MAD) where appropriate (cristalloides; colloides) for each parameter and the *P*-value for comparison between vascular and control trauma population. Significant *P*-values are shown in bold. (normal ranges: hemoglobin: 11.5-16 g/dL; prothrombin time according to Quick: 70–120%; activated partial thromboplastin time, PTT: 25–36 s; fibrinogen: 1.6–4.0 g/L; lactate: < 0.5 mmol/L; Myoglobin: < 55 μg/L; creatin kinase: < 170 U/L)

Subsequent analysis of the pre-clinical fluid administration revealed a significantly higher need of total volume to secure safe transport (Table 1). As expected, the total volume correlated inversely with the initial hemoglobin measurement at admission (*p* < 0.001). Regression analysis revealed lowering of the hemoglobin-value by 1 mg/dl with every 200 ml of fluids administered. This effect was four times stronger in the vascular trauma group (Additional file 3: Table S3). Unfortunately, initial blood pressure measurements and reports about tourniquet or pelvic clamp usage were not available. A drop in fibrinogen by 1 g/L was associated with roughly 700 ml of fluid administration. No significant values were detected in both groups, yet the observed trends suggest a stronger effect with additional vascular damage (Additional file 3: Table S3).

### Outcome analysis

No differences were seen in mortality rates between patients with extremity fracture only and such with associated vascular damage (Table 2). In both groups overall survival was over 90% and death, if any, was due to accompanying head injury, thoracic injury or prolonged course of disease with septic complications. However, a notably high immediate mortality rate (≤24 h) of 13.3% and overall mortality rate of 20% was associated with pelvic fracture with vascular damage. These could, however, not be related to a specific fracture type or vessel involvement (66% arterial/33% venous).

**Table 2:** Outcome parameters and trauma scores of vascular and control trauma populations

	Overall survival	Hospital stay (d)	ICU stay (d)	Operations (number)	Amputation (leg)	GCS	ISS	RISC I
Vascular injury group	92.5%	36.0 ± 22.1	15.0 ± 14.8	6.0 ± 4.7	25.8%	13.2 ± 3.3	23.4 ± 13.8	81.5 ± 25.1
Fracture only group	91.3%	21.5 ± 13	7.2 ± 7.3	2.9 ± 2.4	–	13.3 ± 3.5	22.7 ± 14.2	86.2 ± 23.1
Effect size Effect type	0.486 hazard	12.5 median diff	5.5 median	2	8.32	0	0	−1.9
Test type	ratio Cox regression	Wilcox-Test	diff Wilcox-Test	median diff Wilcox-Test	odds ratio Fisher’s exact-t	median diff Wilcox-Test	median diff Wilcox-Test	median diff Wilcox-Test
*P*	0.28	0.00002	0.009	0.000003	0.03	0.8	0.74	0.3

The table shows the comparison of the vascular trauma group to the fracture only group, indicating mean ± SD, effect size, effect type and the type of test used, along with their significance. Significant *P*-values are shown in bold. (*GCS*,Glasgow Coma Scale, *ISS* Injury Severity Index, *RISC I*,Revised Injury Severity Classification Score)

Fracture-associated vascular disruption resulted in longer hospital stay, longer ICU stay and a higher number of revision surgery after the initial procedure for bleeding control and primary fixation (Table 2). Also, the lower extremity amputation rate was significantly higher. Two amputations were due to primary subtotal above-the-knee amputation during trauma, five were due to severe soft tissue damage and one was due to prolonged ischemia after complicated pelvic fracture with iliac artery occlusion. No upper extremity amputation occurred. The grade of soft tissue damage was, however, comparable for lower and upper extremities (grade IIIC according to Gustilo/Anderson in > 70% of cases). Compartment release had to be done in eight lower and one upper extremity, these cases did not correlate with subsequent need for amputation. These implications were not reflected by either of the trauma scores GCS, ISS or RISC I (Table 2).

Vascular surgical and interventional endovascular means were applied for bleeding control. Open surgical procedures included autologous vein interposition, patch plasty, ligature as well as direct suture and were the method of choice in both extremities (Figs. 1 and 3). Pelvic fracture associated bleedings were mostly treated by endovascular coiling or covered stent implantation in one case (Figs. 1 and 2).

## Discussion

Fracture-associated vascular injury of the limbs and especially the pelvis may have severe consequence for the patient. Accurate detection, early treatment and supportive therapy determine the outcome [15].

Mechanisms of vascular trauma include blunt trauma (external force or through fracture fragments), sharp trauma or strain which might lead to disruption or intima damage leading to ischemia (Fig. 3) [16, 17]. Presentation is thus hemorrhage or ischemia, a phenomenon best described for supracondylar humeral fracture or knee trauma [18–20]. The initial clinical symptoms, especially hemodynamic instability, were not available retrospectively in our cohort. Nevertheless, the established algorithm of local CT-angiography in case of suspected severe fracture of the pelvis and the extremities provided good results, since all patients with vascular injury were identified immediately and no late amputations due to ischemia occurred [5, 12, 21].

In 3–9% of fractures a concomitant vascular damage is reported varying due to regional and socioeconomic factors [10, 22–24]. In this single-center cohort we identified an accompanying vascular damage in 7% of the fractures. Required treatments were very heterogeneous, for the fracture-associated vascular injury as well as vascular incidents in general (Fig. 1). Unfortunately, most publications are case reports or case series focusing on a specific entity or treatment, thus not catering for the need for more general data analysis [25]. No conclusive pattern of injury for consecutive vascular damage could be identified in our cohort, in fact, vascular damage has to be expected regardless of the fracture localization, the impact of trauma or any trauma scores (Table 2).

This data displays a similar incidence of vascular injury in upper/lower extremity and pelvic fractures. Nevertheless, outcomes such as higher immediate mortality in pelvic fractures and significant amputation rates for the lower extremity are area-specific (Fig. 1 and Table 2). The management is considerably influenced by the degree of tissue damage and occasional primary subtotal amputation. Of 15 pelvic fractures, 4 were classified stable while the rest were type B/C fractures (Pennal and Tile classification) possibly yielding additional rupture of the venous plexus. Patients with fractures and vascular injuries also show a longer hospital stay and higher number of operative procedures (Table 2). Elevated immediate mortality in the fracture only group was due to the higher rate of multiple fractures, suggestive of more severely trauma (Table 2 and Additional file 2: Table S2). The additional severity of vascular damage to multi-injured patients is currently not reflected by any trauma score (Table 2).

This study demonstrates the effect of additional vascular damage on the admission laboratory data (Table 1 and Additional file 2:Table S2 and Additional file 3: Table S3). The presented results show similar trends in overall, as well as entitiy-specific analysis (Additional file 2: Table S2). Low hemoglobin, and altered coagulation were, however, associated with an increased need of fluid in the preclinical course. Thus some dilution effect has to be taken into consideration However, the available data in retrospective analysis was not available in all patients, and results have to be interpreted carefully. Nevertheless, substantial fluid administration and low initial hemoglobin, i.e. by rapid blood gas analysis, may raise the attention to direct diagnostics to identifying vascular damage. Trauma-induced coagulation defect is a severe co-morbidity, strongly associated with an increased mortality [26]. Early therapy might also include platelet-directed treatments [27, 28].

Ever since the late stages of World War II, where ligation of arteries and veins was the treatment of choice, surgical and technical abilities have evolved towards a diversified armamentarium, significantly increasing limb salvage and survival rates, nowadays enabling individualized care [29, 30]. Especially rapid endovascular repair for bleeding control has evolved as primary option for certain clinical entities of vascular injury, like embolization in pelvic bleeding, stentgraft implantation in aortic transection or thoracic outlet vessel breach [22, 25, 31, 32]. However, mangled extremities remain a domain of open vascular repair (Fig. 1). Open fracture, eventual contamination and massive tissue loss require autologous reconstruction [9, 33, 34].

## Conclusion

This retrospective single-center cohort study we could demonstrate that fracture-associated vascular damage occurs in one of fourteen trauma patients, with a specific clinical impact regarding pre-hospital fluid management, eventual early transfusion and coagulation support. A multidisciplinary approach requires trauma and vascular surgery in combination with interventional radiology to guarantee the best possible outcome for overall survival and limb salvage.

## Additional files

Additional file 1: **Table S1.** Baseline characteristics of vascular and control trauma populations. Both populations have a similar size and show no differences in age, sex and trauma type distribution. The number of patients with multiple fractures is slightly higher in the control trauma population. (DOC 31 kb)

Additional file 2: **Table S2.** Laboratory results and total preclinical volume comparison between vascular and control trauma population overall and based on area of fracture. The table shows the mean ± SD or median ± MAD where appropriate for each parameter and the *P*-value for comparison between vascular and control trauma population. Significant *P*-values are shown in bold. (normal ranges: hemoglobin: 11.5-16 g/dL; prothrombin time according to Quick: 70–120%; activated partial thromboplastin time, PTT: 25–36 s; fibrinogen: 1.6–4.0 g/L; lactate: < 0.5 mmol/L). (DOC 45 kb)

Additional file 3: **Table S3.** Preclinical fluid administration in dependence of hemoglobin and fibrinogen. Regression results of indicated parameters against total administered volume are shown for the overall study population as well as both patient groups separately. The effect size corresponds to the average volume of required preclinical fluid administration when the examined parameter value is increased by 1. Significant P-values are shown in bold. (DOC 35 kb)

## Abbreviations

CT: Computed tomography
CTA: computed tomography angiography
GCS: Glasgow coma scale
ISS: Injury severity index
PTT: Partial thromboplastin time
RISC I: Revised Injury Severity Classification Score
