# Supplementary material for: Clinical implications of fracture-associated vascular damage in extremity and pelvic trauma
Source: BMC Musculoskelet Disord. 2018 Nov 20;19:404. doi: 10.1186/s12891-018-2333-y (PMC6247697; doi:10.1186/s12891-018-2333-y)
Supplement: Supplementary file 1 — Table S1. Baseline characteristics of vascular and control trauma populations. Both populations have a similar size and show no differences in age, sex and trauma type distribution. The number of patients with multiple fractures is slightly higher in the control trauma population. (DOC 31 kb) [file 12891_2018_2333_MOESM1_ESM.doc]

|  | **N** | **age**  (y) | **sex** | **trauma**  **type** | **% multiple fractures** |
| --- | --- | --- | --- | --- | --- |
| vascular injury  group | 64 | 49±17.5 | 50 m  14 f | 60 blunt  4 penetrating | 39.7% |
| fracture only  group | 60 | 48±20.1 | 39 m  21 f | 58 blunt  2 penetrating | 50.6% |
| P |  | 0.76 | 0.15 | 0.12 | 0.07 |

**supplementary table 1: Baseline characteristics of vascular and control trauma populations.** Both populations have a similar size and show no differences in age, sex and trauma type distribution. The number of patients with multiple fractures is slightly higher in the control trauma population.
