# Supplementary material for: Clinical implications of fracture-associated vascular damage in extremity and pelvic trauma
Source: BMC Musculoskelet Disord. 2018 Nov 20;19:404. doi: 10.1186/s12891-018-2333-y (PMC6247697; doi:10.1186/s12891-018-2333-y)
Supplement: Supplementary file 2 — Table S2. Laboratory results and total preclinical volume comparison between vascular and control trauma population overall and based on area of fracture. The table shows the mean ± SD or median ± MAD where appropriate for each parameter and the P-value for comparison between vascular and control trauma population. Significant P-values are shown in bold. (normal ranges: hemoglobin: 11.5-16 g/dL; prothrombin time according to Quick: 70–120%; activated partial thromboplastin time, PTT: 25–36 s; fibrinogen: 1.6–4.0 g/L; lactate: < 0.5 mmol/L). (DOC 45 kb) [file 12891_2018_2333_MOESM2_ESM.doc]

|  |  | **hemoglobin** (mg/dl) | P | **Quick**  (%) | P | **PTT**  (s) | P | **fibrinogen**  (g/l) | P | **lactate**  (mmol/l) | P | **total volume**  (ml) | P |
| --- | --- | --- | --- | --- | --- | --- | --- | --- | --- | --- | --- | --- | --- |
| vascular injury | overall | 10±2.6 |  | 59±25.2 |  | 41.9±21.0 |  | 1.7±0.9 |  | 2.1±1.2 |  | 1500±1111 |  |
| group | upper limb | 11.1±2 | vs | 80±19.3 | vs | 30.3±7.0 | vs | 2.3±0.2 | vs | n.a. | vs | 1250±0 | vs |
|  | pelvic | 10.1±2.9 |  | 44±15.6 |  | 58.7±32.1 |  | 1.1±0.4 |  | n.a. |  | 1500±1111 |  |
|  | lower limb | 9.3±2.5 |  | 58±20 |  | 40.3±13.7 |  | 1.6±0.9 |  | n.a. |  | 2250±1111 |  |
| fracture only | overall | 11.6±2.6 | **0.0004** | 78±19.3 | **0.0002** | 35.3±20.7 | **0.002** | 2.2±0.6 | **0.003** | 1.8±0.9 | 0.24 | 1000±741 | **0.03** |
| group | upper limb | 11.4±2.3 | 0.69 | 79±20.1 | 0.9 | 29.0±6.0 | 0.42 | 2.4±0.7 | 0.68 | n.a. |  | 1500±741 | 0.86 |
|  | pelvic | 11.1±2.3 | 0.35 | 65±24.5 | 0.22 | 41.4±18.6 | 0.07 | 1.9±1.0 | 0.07 | n.a. |  | 1000±0 | 0.1 |
|  | lower limb | 12±2.9 | **0.0002** | 82.5±14.1 | **0.00005** | 33.0±15.4 | **0.03** | 2.2±0.4 | **0.02** | n.a. |  | 1125±926 | **0.02** |

**supplementary table 2: Laboratory results and total preclinical volume comparison between vascular and control trauma population overall and based on area of fracture.** The table shows the mean ± SD or median ± MAD where appropriate for each parameter and the P-value for comparison between vascular and control trauma population. Significant P-values are shown in bold. (normal ranges: hemoglobin: 11.5-16g/dL; prothrombin time according to Quick: 70-120%; activated partial thromboplastin time, PTT: 25-36s; fibrinogen: 1.6-4.0g/L; lactate: <0.5mmol/L)
