# Supplementary material for: Clinical implications of fracture-associated vascular damage in extremity and pelvic trauma
Source: BMC Musculoskelet Disord. 2018 Nov 20;19:404. doi: 10.1186/s12891-018-2333-y (PMC6247697; doi:10.1186/s12891-018-2333-y)
Supplement: Supplementary file 3 — Table S3. Preclinical fluid administration in dependence of hemoglobin and fibrinogen. Regression results of indicated parameters against total administered volume are shown for the overall study population as well as both patient groups separately. The effect size corresponds to the average volume of required preclinical fluid administration when the examined parameter value is increased by 1. Significant P-values are shown in bold. (DOC 35 kb) [file 12891_2018_2333_MOESM3_ESM.doc]

|  |  | **effect size** (ml) | **P** |
| --- | --- | --- | --- |
| hemoglobin | overall | -211.1 | **0.0005** |
|  | vascular injury group | -316.3 | **0.002** |
|  | fracture only group | -66.3 | 0.43 |
| fibrinogen | overall | -709.5 | **0.01** |
|  | vascular injury group | -692.3 | 0.08 |
|  | fracture only group | -330.5 | 0.13 |

**supplementary table 3: Preclinical fluid administration in dependence of hemoglobin and fibrinogen.** Regression results of indicated parameters against total administered volume are shown for the overall study population as well as both patient groups separately. The effect size corresponds to the average volume of required preclinical fluid administration when the examined parameter value is increased by 1. Significant P-values are shown in bold.
